# The relationship between high school English learners’ anxiety and their smartphone addiction: evidence from a daily diary approach

**DOI:** 10.3389/fpsyt.2025.1581404

**Published:** 2025-07-30

**Authors:** Chengli Zhang, Ying Han, Bingqiang Li, Jing Wang

**Affiliations:** ^1^ Department of Foreign Languages, Xinzhou Normal University, Xinzhou, China; ^2^ Department of Tourism and Management, Xinzhou Normal University, Xinzhou, China; ^3^ School of Education, Changzhou Institute of Technology, Changzhou, China; ^4^ Department of Education, Xinzhou Normal University, Xinzhou, China

**Keywords:** anxiety, smartphone addiction, daily diary approach, English learner, high school

## Abstract

**Background:**

The issue of smartphone addiction among high school English learners is becoming more and more significant in this era of information technology, and it is directly associated with their anxiety.

**Methods:**

To examine the correlation between high school English learners’ anxiety level and the severity of smartphone addiction, the anxiety and smartphone addiction levels of high school English learners were explored. SPSS 26 and MPLUS 8.3 were utilized for data analysis. The sample selected by stratified random sampling comprised 248 high school English learners from a high school in Shanxi Province, and a diary survey was conducted for seven consecutive days, obtaining a total of 1,610 valid data points.

**Results:**

The findings of the multilevel regression model indicated a substantial correlation between anxiety level and the severity of smartphone addiction among high school English learners. Anxiety level was a substantial favorable indicator of the severity of smartphone addiction; a notable disparity existed in the smartphone addiction levels of males and females; however, no significant difference was observed between the genders regarding the predicted influence of anxiety level on the severity of smartphone addiction.

**Conclusions:**

This study examined the effect of anxiety levels on the severity of smartphone addiction and analyzed whether there were gender differences. Based on the results, this study proposes a study on anxiety and smartphone addiction interventions for high school English learners in basic education. The proposal offers evidence from both empirical and theory-based investigations to substantiate the healthy development of high school English learners.

## 1 Introduction

With the widespread use of smartphones, they have become an important tool for language learning (1, 2). For English learners, audiovisual materials on smartphones can help learners immerse themselves in real-life environments (3, 4), thereby enhancing their English proficiency (5). While smartphones have numerous positive effects on English learning, it cannot be denied that they also have negative impacts on learning (6). As reliance on smartphones continues to grow (7), the risk of smartphone addiction(SA) inevitably increases (8), particularly among adolescents (9–11).

Mark D Griffiths (12) argues that smartphone addiction is a form of behavioral addiction. This addiction exhibits characteristic features of addiction, including pleasurable sensations, tolerance, withdrawal symptoms, and relapse (12, 13). Smartphone addiction can lead users to become excessively obsessed with smartphone use, resulting in intense and persistent cravings and dependence, which may harm their psychological and social functioning (14). Currently, gender differences in SA symptoms have become one of the key areas of focus for researchers. Research has not reached a consensus on whether men or women are more prone to SA (15–19). However, regardless of the results, it is undeniable that gender has become a factor that must be considered in analyzing SA.

Research indicates that there may be numerous factors contributing to SA symptoms. For example, personality traits (20), emotional and cognitive needs (21, 22), and habitual smartphone use (23). Anxiety(AN), as a common negative emotion (24), is considered an important contributing factor to SA symptoms. AN primarily manifests as uncontrollable worry and fear (25, 26), which may lead to physical illnesses, social difficulties, academic burnout, and other physiological or psychological issues (27–29), particularly prevalent among adolescents (30, 31), and foreign language learners are no exception (32). This study treats AN symptoms as a negative emotion.

To alleviate AN symptoms, many adolescents overuse smartphones to distract themselves. A link has been established between AN and SA symptoms (33–35). Smartphones, as tools for social interaction, information retrieval, and entertainment and relaxation, can effectively alleviate AN symptoms in learners (36–38). However, while most researchers have extensively studied AN and SA among students of different age groups (39–41), However, studies systematically collecting data using continuous observation diary methods for this group are scarce, and few studies have conducted in-depth explorations of the relationship between these two variables using multilevel regression models.

Therefore, this study aims to use diary methods and data model analysis to explore the complex relationship between AN among middle school English learners and SA, as well as the impact of gender differences. Based on the research objectives and theoretical framework, the following hypotheses were formulated:

H1: The severity of SA among high school English learners has gender differences.
H2: High school English learners’ AN positively predicts the severity of SA.

## 2 Methodology

### 2.1 Participants

The data for this study were obtained from a broader research initiative, employing random stratified sampling in Shanxi Province, China, with a total number of 248 senior high school students. After data collection, the researcher checked the validity of the completed questionnaires; total valid questionnaires were 230. Here is the formula to determine the sample size:


$$n=\frac{Z^{2}p(1-p)}{e^{2}}$$

n is the sample size, Z is the standard error, p is the variance of the population estimate, and e is the permissible error.

When the 95% confidence interval, Z is 1.96. p is taken to be 0.5 for prudent estimation. The permissible error is 0.01. The formula calculation shows that at least 97 participants are needed for this study. Sample size is higher than 97. The study participants consisted of high school students who selected Physics, Chemistry, and Biology for the National College Entrance Examination to pursue admission in General Higher Education. The participants were teenagers aged 15 to 18 years (mean age = 16.4); 112 (48.70%) were male, and 118 (51.30%) were female. The sample comprised 160 (69.57%) students who lived in urban areas and 70 (30.43%) in rural areas. Furthermore, informed agreement was obtained from both the students and their parents before to the experiment, and the study’s goal was elucidated to the participants. The participants agreed to providing anonymized data for the current study.

### 2.2 Procedure

This study had three principal phases. During the initial phase, participants signed a written informed permission form and completed a questionnaire intended to gather demographic and baseline data. Those who finished the questionnaire and supplied their assent were eligible to participate in a diary tracking survey. In the second phase, the diary tracking survey was conducted for seven consecutive days using a paper diary checklist for data collection. The collected data provided demographic information, including details of gender, date of birth, grade level, family residence, and parental occupation. The baseline questionnaire and diary inventory consisted of scales pertaining to SA and AN. Data of all participating students were finally collected on June 15, 2024, with daily diary reports on campus between 17:30-18:00 pm. Reports were submitted daily. Finally, the responses to the diary questionnaire were recorded by the researcher.

### 2.3 Measures

#### 2.3.1 Daily anxiety scale

The Negative Mood Scale proposed by Clark and Watson (42) and compiled by Antony et al. (43), which includes three dimensions: AN, depression, and stress, was used in the study; it has 21 items, but, in this study, only the subscale “AN” was considered. After discussion among the authors, some of the wording was modified to accommodate the diary measurements (e.g. “I don’t seem to feel any pleasantness or relief at all today”). Participants self-assessed utilizing a 4-point Likert scale, with 1 indicating ‘does not meet’ and 4 denoting ‘always meets.’ This study reported Cronbach’s alpha coefficients for the scale ranging from 0.890 to 0.967 for each day, with an overall coefficient of 0.945 throughout the 7 days.

#### 2.3.2 Daily Smartphone Addiction Scale

The Smartphone Addiction Scale-Short Version (SAS-SV), developed by Kwon et al. (44), is a short version of the Smartphone Addiction Scale, which consists of 10 items (e.g., “I will change my study program today because of using my smartphone”). Following deliberation, certain phrasing was altered to align with the diary measure. Participants evaluated themselves utilizing a Likert scale, with responses spanning from 1 (strongly disagree) to 6 (strongly agree). In the present research, the Cronbach’s alpha coefficients for the scale varied from 0.900 to 0.977 daily, yielding a cumulative Cronbach’s alpha coefficient of 0.961 throughout the 7 days.

### 2.4 Data analyses

The data of this study had a nested structure, i.e., the daily survey data of the study participants (within-individual level, Level 1) were nested within the overall data of the individuals (between-individual level, Level 2). Therefore, a multilevel model was required for data visualization.

The data was processed with SPSS 26.0 and Mplus 8.3. First, data was collected using the self-reporting technique, and in order to avoid common method bias due to subjective reporting, Harman’s one-factor test was employed to assess common method biases (CMB). Second, descriptive statistics were analyzed for all samples to understand the sample composition. Additionally, the relationship among AN and dependency on smartphones was analyzed utilizing Pearson’s coefficient. to explain the relationship between variables more scientifically. In addition, Mplus8.3 was used to determine the intra-class correlation coefficient (ICC) for each variable to authenticate the suitability of the data in this research for multilevel evaluation. At last, Mplus 8.3 was additionally employed to develop a multilevel regression model to test the relationship between AN level and the severity of SA.

## 3 Results

### 3.1 Common method biases analysis

To verify the data’s reliability, the CMB was determined utilizing the Harman one-way test prior to data processing. By testing the 17 items in the questionnaire related to the two variables, the results showed that the three factors had eigenvalues greater than 1, and the maximum factor variance explained was 38.717%, which did not reach the critical value of 40%, and hence, no serious common variance bias was found (45).

### 3.2 Descriptive statistics and correlation analysis

The outcomes of descriptive statistical methods and correlation analysis between AN level and the severity of SA among students are displayed in **Table 1**. **Table 1** illustrates that there are 230 responders., the mean value of SA in high students was 3.78 with a standard deviation of 0.72 and an intra-individual standard deviation of 0.94. The mean value of AN was 3.92 with a standard deviation of 0.66 and an intra-individual standard deviation of 0.86.

**Table 1:** Descriptive statistics and correlation analysis.

Variables	M (SD)	SDwp	Smartphone addiction	Anxiety
Smartphone Addiction	3.78 (0.72)	0.94	1	.541**
Anxiety	3.92 (0.66)	0.86	.478**	1

** Denotes p < 0.01. M denotes mean and SD_wp_ denotes standard deviation among individuals. The correlation coefficient at Level 1 (N = 1610) is below the diagonal, whereas the correlation coefficient at Level 2 (N = 230) is above it.

Pearson’s product-moment relationship analysis was employed to investigate the correlations among the model parameters at Level 1 and Level 2, with correlation coefficients at Level 2 above the diagonal line (N = 230) and correlation coefficients at Level 1 below the diagonal line (N = 1610). It was found that AN level had a substantial positive correlation with the severity of SA (r = 0.478, p < 0.01).

### 3.3 Intra-class correlation coefficient analysis

ICC is the ratio of interindividual variation to total variation and indicates the proportion of all variation explained by the interindividual variation. In this study, the null model was used to calculate the intragroup correlation coefficient for each variable (46).

**Table 2** indicates that the ICC for SA was 0.616, indicating that 61.6% of the variation came from inter-individual differences and 38.4% of the variation came from intra-individual differences. The ICC for AN was 0.593, indicating that 59.3% of the values were attributed to the inter-individual variation and 40.7% of the values were attributed to the intra-individual variation. The ICC values for all variables were greater than 0.059, indicating that within-group similarities could not be ignored, thus allowing for multilevel analysis (47).

**Table 2:** Intra-group correlation coefficients.

Variables	Smartphone addiction	Anxiety
ICC	0.616	0.593

### 3.4 Regression analysis

Using students’ gender as a moderator, the relationship between AN and SA was investigated. After determining the Level 1 predictive effect of AN on SA, the following model was built:


$$Y_{ij}=\beta_{0j}+\beta_{1j}X_{ij}+\epsilon_{ij}$$

Where $\epsilon_{ij}∼N\;\left(0,\;\sigma^{2}\right)$ .

The differences between males and females at different levels of SA and the predictive effect of AN on SA were determined through Level 2, using students’ gender as a predictor variable. The modeling constructs are as follows:


$$\beta_{0j}=\gamma_{00}+\gamma_{01}W_{j}+\mu_{oj}$$

$$\beta_{1j}=\gamma_{10}+\gamma_{11}W_{j}+\mu_{1j}$$

Where $\left[\begin{array}{c} \mu_{oj}\\ \mu_{1j} \end{array}\right]∼N\left[\left[\begin{array}{c} 0\\ 0 \end{array}\right],\;\left[\begin{array}{cc} \tau_{00} & \tau_{01}\\ \tau_{10} & \tau_{11} \end{array}\right]\right]$ .

Let $Var\left(\beta_{0j}\right)=Var\left(\mu_{0j}\right)=\tau_{00}$ , $Var\left(\beta_{1j}\right)=Var\left(\mu_{1j}\right)=\tau_{11}$ , $Cov\left(\beta_{0j},\;\beta_{1j}\right)$ = $\tau_{01}$ .

In this model, the intercept and slope of Level 1 are determined by the Level 2 factor, i.e., all changes in Level 1 are reflected in Level 2, and the variance exists in Level 2. Therefore, Level 1 results only output the residual variance (σ2), which is valued at 0.240. Male students had a SA level of 1.175, according to the results shown in **Table 3**, and there was a substantial difference between male and female students in terms of SA (β = 0.906, SE = 0.356, p < 0.05). AN level in males was a significant positive predictor of SA (β = 0.552, SE = 0.136, p < 0.001), but the predictive effect of AN on SA of males and females did not differ significantly (β = -0.158, SE = 0.086). That is, there was no gender difference in the predictive effect of anxiety level on the severity of smartphone addiction. The overall variance of each student’s AN was 3.364, and the overall variance of each student’s slope was 0.192. Thus, males and females’ levels of SA differed significantly from one another; additionally, males’ and females’ levels of AN significantly and positively predicted the severity of SA.

**Table 3:** Multi-level regression results.

Variables	Estimated value	SE
Fixed effect		
Intercepts (ϒ_00_)	1.175*	0.564
Gender (ϒ_01_)	0.906*	0.356
Anxiety (ϒ_10_)	0.552***	0.136
Anxiety*Gender (ϒ_11_)	-0.158	0.086
Random effect		
Intercepts (τ_00_)	3.364***	0.603
Anxiety (τ_11_)	0.192***	0.036
Residual (σ^2^)	0.240***	0.010
Model fit		
N	1610	
LL	-1500.073	
AIC	3016.147	
BIC	3059.219	

*p < 0.05, ***p < 0.001.

## 4 Discussion

### 4.1 Discussion of results

As smartphones have become an integral part of life, SA among adolescents has become a challenging public health issue today. This study used a diary method to explore the role of anxiety levels of high school English learners in influencing their SA symptoms and whether there were gender differences. The results of the study indicated that anxiety level of high school English learners was a predictor of smartphone addiction severity. There was a significant difference in the level of smartphone addiction between males and females, but no significant difference between the genders was observed in the predictive effect of anxiety level on the severity of smartphone addiction. The findings contribute to a deeper understanding of the relationship between anxiety levels and the severity of SA among high school English language learners.

First, the results of this study are consistent with H1 that there is a significant gender difference in the severity of SA among high school English learners. It has been shown that the difference in SA symptoms between genders is statistically significant (48–50). The possible reason for this is that males and females have differences in smartphone app usage. Among them, males prefer mobile games while females favor social media applications (51). This reflects the fact that females have more need to communicate and maintain relationships through smartphones for socialization purposes (52, 53). Therefore, if SA symptoms are to be reduced among high school English learners, educators need to take into account the gender differences of students.

Second, the study confirmed that H2, the daily anxiety level of both males and females, positively predicted their daily SA symptoms. On the one hand, high school English learners have foreign language anxiety such as reading, writing, and listening anxiety in English learning, which can cause students to develop negative emotions (54, 55). According to the negative reinforcement emotional processing model, it is known that high school English learners develop addictive behaviors in order to escape or reduce the negative emotions caused by English anxiety (56). Smartphones are characterized by instant gratification, easy accessibility, and attention demand (57), which can provide individuals with diversified entertainment and effectively ease their anxiety (58), increasing the likelihood of students’ SA. When a student’s need to alleviate anxiety is met, he or she will continue to use the smartphones more frequently (59). On the other hand, in the field of English language learning, female English language learners are more prone to English anxiety due to greater emotional sensitivity compared to males (60), but females are more adept at language learning and language use (61). This may alleviate females’ lack of emotional control over English anxiety. Therefore, there was no significant difference between high school English language learners in the relationship between anxiety levels and the severity of SA. It should be noted that the present study only examined whether there were gender differences between anxiety levels on SA symptoms among high school English learners, and due to the limited data, it was not possible to discuss whether gender differences would have an impact on the correlation between single anxiety items in foreign languages, such as listening, speaking, reading, and writing, and SA, which could be explored in future studies.

### 4.2 Implications

This study offers distinctive insights on SA with significant theoretical and practical ramifications for frontline teachers. In terms of theoretical implications, this study was unique in that it explored the effect of anxiety levels on the severity of smartphone addiction and analyzed whether there were gender differences. In terms of practical implications, this study provides targeted insights for preventing smartphone addiction due to anxiety in language learners. Faced with anxiety during language learning, males will tend to use relaxation as a way of coping, while females tend to seek companionship and think positively (62). Therefore, teachers can alleviate the symptoms of smartphone addiction in students according to their gender differences. Teachers can also choose topics that students enjoy in class, using slower speech, simple words and grammar, thus easing the severity of smartphone addiction by reducing anxiety. This in turn encourages students to engage in self-talk and active participation in the classroom in English (63). In addition, educational researchers can improve the excessive use of smartphones by students from a psychological point of view. For example, teachers can enhance communication with students to understand the source of their AN and help them overcome their SA by alleviating their anxiety levels.

### 4.3 Limitations and suggestions for future research

Some restrictions apply to this research. First, all participants in this study were sophomores from a school in Shanxi Province, China, and hence the findings cannot be generalized. Future research can select students from other districts for the study. Second, this study proved that there is a significant gender difference in students’ smartphone addiction, but the size of the gender difference was not clarified due to the limitations of the research methodology. In addition, the number of topics that participants had to report in this diary study was high, which may have affected their motivation to participate in the study and hence affected the reliability of the questionnaire. In the future, we will improve this study in several ways. First, we will seek a more comprehensive research methodology to clarify the magnitude of specific gender differences between language learner anxiety and smartphone addiction. Second, researchers can we will expand the scope of the study by recruiting participants from different schools in different districts and even from different school years. In addition, researchers could explore the relationship between the dimensions of anxiety and other constructs as a way to more fully explore the relationship between anxiety and smartphone addiction. While the relationship between AN level and the severity of SA may still be controversial, this study could provide future researchers with some insights and necessary evidence.

## 5 Conclusions

In order to investigate the relationship between AN and SA, this study utilized an intensive longitudinal diary method research design to follow high school English learners for seven consecutive days. The findings revealed that AN level was a strong positive predictor of the severity of SA and that there was a substantial difference in the degree of SA between males and females.

## Data Availability

The raw data supporting the conclusions of this article will be made available by the authors, without undue reservation.
